# Analysis of the relevant factors for corneal graft rejection in the southern Liaoning region from 2019 to 2023

**DOI:** 10.3389/fmed.2024.1517198

**Published:** 2025-01-08

**Authors:** Chunxiao Yan, Zhijian Zhang, Lin Jin, Mengxin Liu, Tianyi Wang, Jinghao Yang, Lijun Zhang

**Affiliations:** ^1^Dalian Medical University, Dalian, China; ^2^Department of Ophthalmology, The Third People’s Hospital of Dalian Affiliated to Dalian Medical University, Liaoning Provincial Key Laboratory of Cornea and Ocular Surface Diseases, Liaoning Provincial Optometry Technology Engineering Research Center, Dalian, China; ^3^Jinzhou Medical University, Jinzhou, China

**Keywords:** corneal blindness, keratoplasty, corneal graft rejection, neovascularization, virus keratitis

## Abstract

**Background:**

The study aimed to review the etiology of corneal blindness and investigate the relative risk of corneal graft rejection (CGR) in the southern Liaoning region.

**Methods:**

The clinical records of 359 patients (394 eyes) who underwent corneal transplantation at the Department of Keratoconus of the Third People’s Hospital of Dalian from January 2019 to December 2023 were retrospectively analyzed. The data included patients’ age, gender, occupation, diagnosis, surgical procedure, postoperative immune rejection, and neovascularization. The data were collected and descriptively analyzed to characterize the etiology of corneal blindness and to analyze the risk factors for postoperative immune rejection after corneal transplantation using logistic regression.

**Results:**

The mean age of the patients who underwent corneal transplantation was 55.90 ± 0.80 years, and there were more male patients than female patients with corneal blindness. Infectious keratitis (41.1%) was reported as the leading cause of corneal blindness, and penetrating corneal transplantation was the main surgical procedure for the recovery of sight. Preoperative corneal vascularization and penetrating corneal graft rejection were identified as risk factors for immune rejection of corneal grafts. The preoperative corneal vascularization was performed (*p* = 0.044, OR = 2.607). Penetrating keratoplasty (PKP) was performed (*p* = 0.024, OR = 1.953), and deep anterior lamellar keratoplasty was also performed (*p* = 0.801, OR = 1.088). Viral infections (*p* < 0.001, OR = 16.871) were the major risk factor for preoperative corneal neovascularization (CNV) compared to other etiologies, such as fungal infections (*p* < 0.001, OR = 0.018), mechanical ocular trauma (*p* < 0.001, OR = 0.034), immune keratitis (*p* = 0.023, OR = 0.152), and endothelial dysfunction (*p* < 0.001, OR = 0.054).

**Conclusion:**

Infectious keratitis was identified as the major cause of corneal blindness in the southern Liaoning region over the past 5 years. Penetrating keratoplasty and preoperative corneal vascularization were the risk factors for corneal graft rejection. In addition, virus-derived keratitis was considered to be the main risk factor for corneal neovascularization, and deep anterior lamellar keratoplasty was not found to have an effect on corneal graft rejection in this study.

## Introduction

Corneal blindness is the fifth most common form of blindness worldwide, followed by cataract, glaucoma, refractive error, and age-related cataracts (1). Corneal blindness is a complex condition with various etiological factors, including infections, immuno-inflammatory responses, trauma, keratoconus, and corneal degeneration, all of which often lead to corneal scarring and vascularization, resulting in functional blindness (2). Infectious keratitis remains the leading cause of corneal blindness in developing countries, and corneal transplantation is the primary method for restoring vision (3). However, the impact of immune rejection after corneal transplantation is significant, with a relatively high rate of graft rejection reported during the postoperative period of 1–3 years, which varies depending on the region. In recent years, there has been a gradual shift toward the understanding that vascularization is a risk factor for corneal graft rejection (CGR), but substantial clinical data supporting the effect of vascularization on graft rejection are still lacking. In this study, we reviewed the etiology of patients who underwent keratoplasty in the southern Liaoning region over the past 5 years and analyzed the related risk factors for corneal graft rejection.

## Patients

This study was approved by the Ethics Committee of the Third People’s Hospital of Dalian and conformed to the Declaration of Helsinki. A retrospective review was conducted to collect the number of keratoplasty procedures performed at the Department of Keratopathy, the Third People’s Hospital, Dalian, from 1st January 2019 to 14th November 2023. Demographic data and information about indications for surgery, surgical techniques, and corneal graft rejection were also collected.

## Data collection and analysis

The consultation information of 359 patients (394 eyes), including demographic details, surgical indications, surgical procedures, and postoperative follow-up records of keratoplasty, was retrieved from the medical record system of the Third People’s Hospital of Dalian. Preoperative corneal pathological transformations and postoperative corneal graft immune rejection were noted. Statistical analyses were performed using SPSS 25.0 software (SPSS Inc., Chicago) and RStudio (Posit, Boston). Categorical data were presented as percentages, and comparisons between the groups were performed using the chi-square test. Measurement data with a non-normal distribution were presented as the median, and the Wilcoxon rank sum test was performed to assess the differences between the groups. Measures with a normal distribution were presented as mean ± standard deviation, and the *t*-test was performed to assess the differences between the groups. Univariate and multifactorial logistic regression analyses were conducted to analyze the influence of corneal graft rejection and corneal neovascularization (CNV), with the odds ratio (OR) and 95% confidence interval (CI) calculated. A *p*-value of less than 0.05 indicated that the differences between the groups were statistically significant.

The methods (4, 5) used for variable selection in the regression analysis were as follows:

1. Univariate logistic regression analysis: Each potential predictor (including age, gender, etiology, and CNV) was individually assessed using univariate analysis to determine its association with the outcome variable (CGR).
2. Variable selection: Variables found to be statistically significant (e.g., *p* < 0.05) in the univariate analysis were included in the multivariate logistic regression model. In this study, the virus variable demonstrated a significant association in the univariate analysis, and age and gender were also included as covariates due to their recognized importance as influential factors.
3. Multivariate logistic regression analysis: The significant variables identified, along with the covariates, were entered into the multivariate model to evaluate their independent effects on the outcome variable, after adjusting for other factors.
4. The collinearity check for the multivariate regression analysis is presented as Supplementary data.

## Results

### The etiology characteristics of corneal blindness in the southern Liaoning region

**Figure 1:**
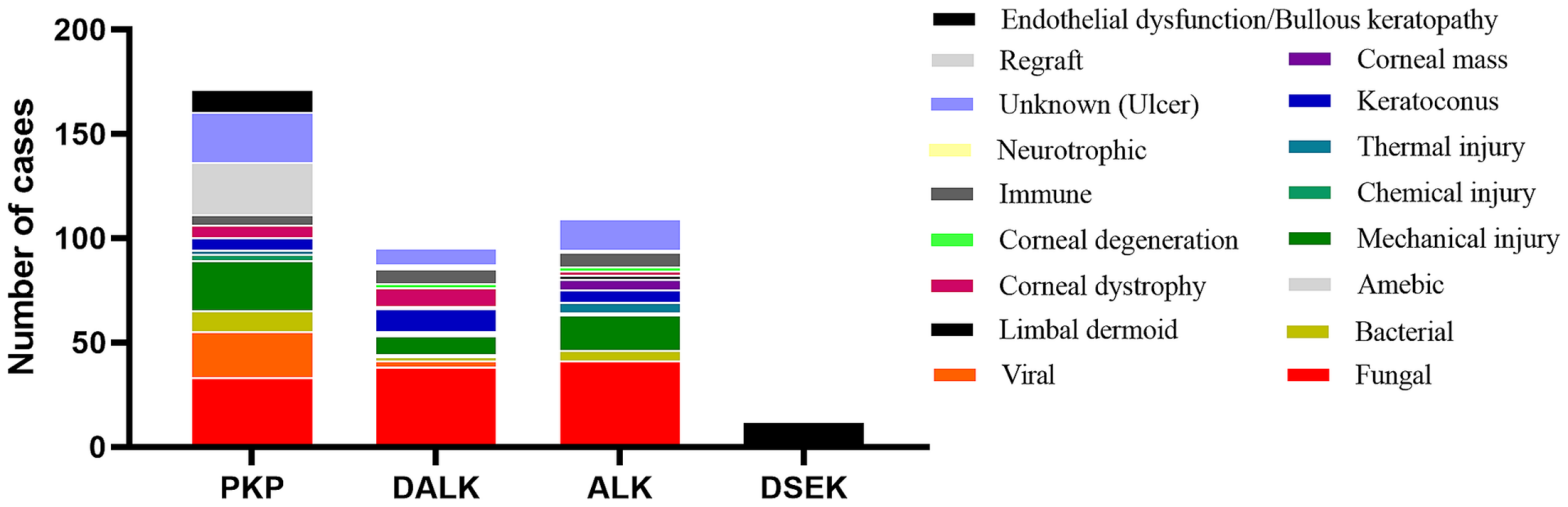
Distribution of the etiologies of keratoplasty from 2019 to 2023. PKP, penetrating keratoplasty, in which the entire layer of the diseased cornea (including the endothelial cell layer) is removed and replaced by a healthy donor cornea. DALK, deep anterior lamellar keratoplasty, in which the entire stromal layer of the diseased cornea is removed and Descemet’s membrane is left intact. ALK, anterior lamellar keratoplasty, in which the superficial stromal layer of the diseased cornea is removed and Descemet’s membrane and the endothelial cell layer are left intact. DSEK, Descemet stripping endothelial keratoplasty, in which Descemet’s membrane and the endothelial cell layer are removed and only corneal Descemet’s membrane and the endothelial cell layer are implanted. All the surgical procedures are standardized.

A total of 359 patients (394 eyes) were included in the retrospective analysis, comprising both male and female participants, with a mean age of 55.90 ± 0.80 years. Among them, 171 underwent penetrating keratoplasty (PKP), 95 underwent deep anterior lamellar keratoplasty (DALK), 116 underwent anterior lamellar keratoplasty (ALK), and 12 underwent Descemet stripping endothelial keratoplasty (DSEK) (Figure 1). The analysis of the etiology of corneal blindness showed that the male participants (68.6%) and individuals without a job (38.3%) were the largest groups of patients, followed by agricultural workers (27.4%). Infectious keratitis (41.1%) was the leading cause of blindness-inducing keratopathy in this region, including fungal keratitis and viral keratitis, followed by ocular trauma (16%), keratoconus (5.8%), and immune corneal diseases (5.1%) (Table 1).

**Table 1:** Classification of the indicators for corneal transplantation.

Characteristic	Total *n* (%)	PKP	DALK	ALK	DSEK
Gender					
Men	260 (66)	118	56	78	8
Women	134 (34)	53	39	38	4
Occupation					
Agricultural workers	108 (27.4)	44	25	36	3
Jobless	151 (38.3)	66	42	40	3
Retiree	61 (15.5)	24	12	21	4
Outdoor worker	41 (10.4)	19	10	11	1
Office worker	11 (2.8)	6	1	4	/
Self-employed person	10 (2.5)	7	/	2	1
Student	12 (3.0)	5	5	2	/
Corneal infections	162 (41.1)	65	44	53	/
Fungal	112	33	38	41	/
Viral	32	22	3	7	/
Bacterial	17	10	2	5	/
Amebic	1	/	1	/	/
Traumatic	63 (16.0)	29	11	23	/
Mechanical injury	50	24	9	17	/
Chemical injury	5	3	1	1	/
Thermal injury	8	2	1	5	/
Keratoconus	23 (5.8)	6	11	6	/
Corneal mass	5 (1.3)	/	/	5	/
Congenital	20 (5.1)	6	10	4	/
Limbal dermoid	3	/	1	2	/
Corneal dystrophy	17	6	9	2	/
Corneal degeneration	4 (1.0)	/	2	2	/
Non-infectious keratitis	20 (5.1)	5	8	7	/
Immune	19	5	7	7	/
Neurotrophic	1	/	1	/	/
Unknown (Ulcer)	47 (11.9)	24	8	15	/
Regraft	27 (6.9)	25	1	1	/
Endothelial dysfunction/Bullous keratopathy	23 (5.8)	11	/	/	12

### Analysis of the factors associated with immune rejection after corneal transplantation

**Figure 2:**
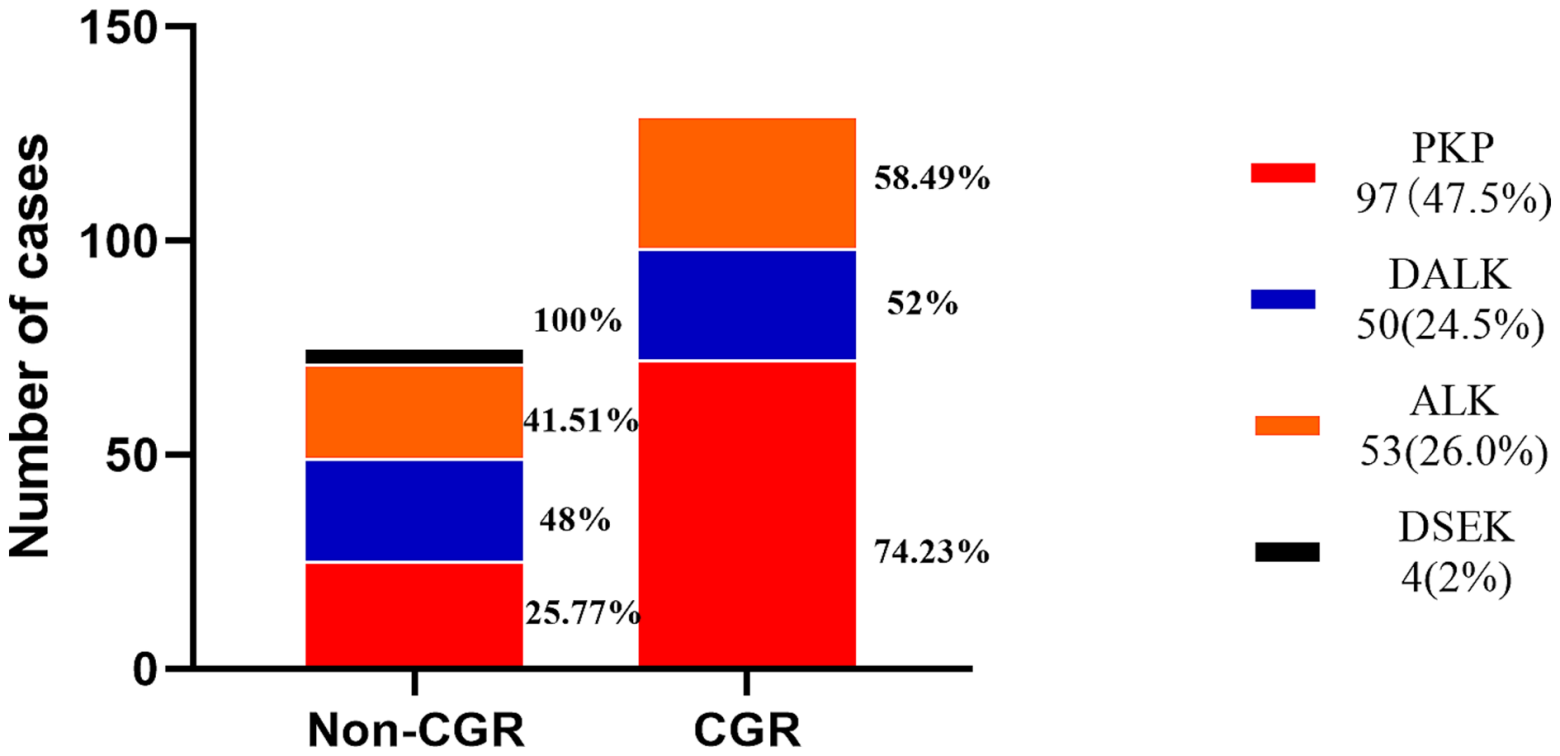
Distribution of the surgical procedures for corneal graft rejection.

**Figure 3:**
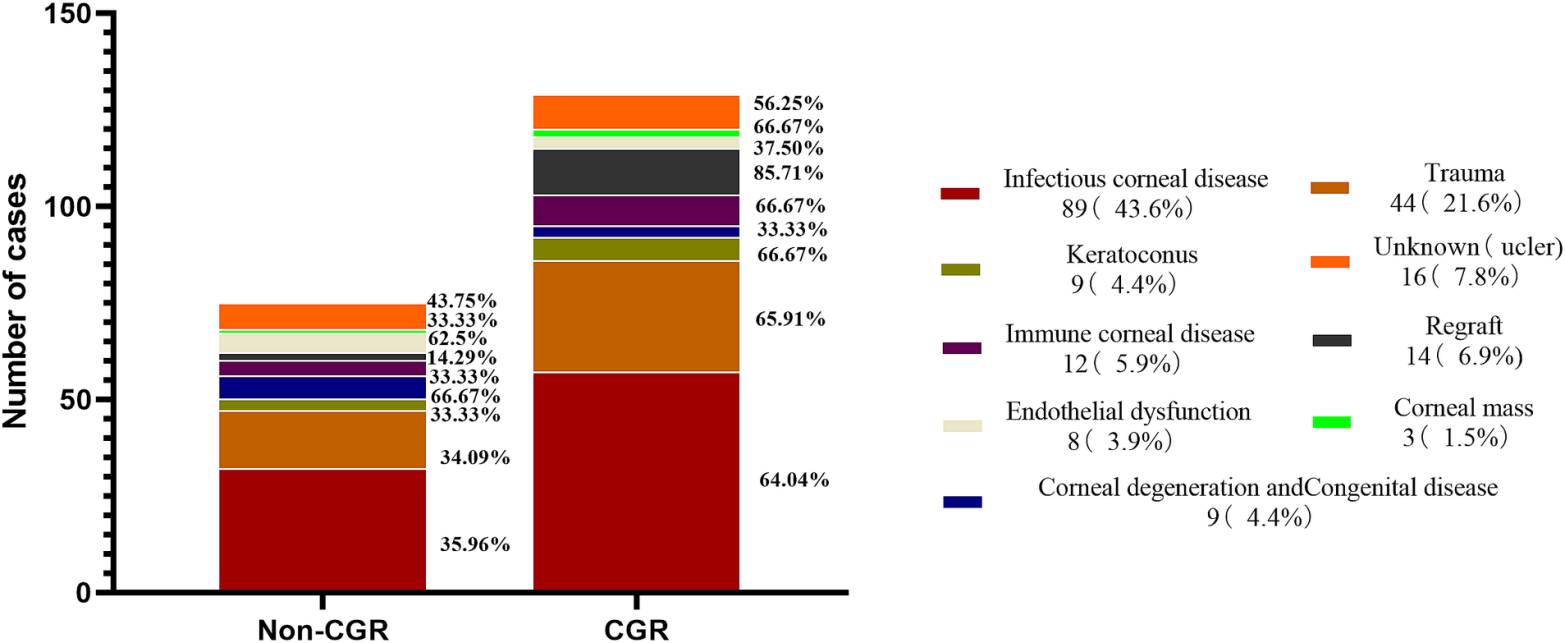
Distribution of the etiologies in corneal graft rejection.

The earliest corneal graft rejection occurred 10 days postoperatively, and the latest rejection occurred 30 months postoperatively. Corneal graft haze, edema, vascularization, and graft resolution were considered indicators of corneal graft rejection. Notably, 209 patients (Han nationality) were included in the statistical analysis. Preoperative CNV (*p* = 0.029) and the graft surgical procedure (*p* = 0.002) were identified as risk factors for corneal graft rejection (Table 2). Further logistic regression analysis of postoperative corneal graft rejection was performed, and PKP was statistically significantly different in the univariate analysis (*p* = 0.024, OR = 1.953, 95% CI 1.103–3.546) and remained statistically significantly different in the multivariate analysis (*p* = 0.046, OR = 1.958, 95%CI 1.013–3.786). There was no evidence of risk of corneal graft rejection with DALK in the univariate analysis (*p* = 0.801, OR = 1.088, 95%CI 0.565–2.115) and multivariate analysis (*p =* 0.962, OR = 0.983, 95%CI 0.490–1.973) (Table 3 and Figure 2). There were 110 cases of stromal edema and haze, 18 cases of corneal implant dissolution, 31 cases of neovascularization, five cases of recurrent infection, and one case of epithelial defect. There were 12 cases of graft rejection due to viral keratitis (12/32), 38 cases of fungal keratitis (38/112), eight cases of bacterial keratitis (8/17), six cases of keratoconus (6/23), and 22 cases of trauma (22/63) (Figure 3). Neovascularization is the most serious postoperative rejection, greatly affecting vision. Our univariate analysis of the risk factors for preoperative corneal neovascularization in relation to postoperative graft rejection found that preoperative CNV significantly increased the risk of postoperative development of CNV (*p* = 0.044, OR = 2.607, 95%CI 0.986–6.562). This difference remained statistically significant in the multivariate analysis of the effects of gender and age (*p* = 0.046, OR = 2.608, 95%CI 1.016–6.695) (Table 4).

**Table 2:** The differences in the distribution of corneal blindness.

Characteristic	Total *n* (%)	Non-CGR	CGR	*χ* ^2^	*p*
Gender				1.957	0.162
Men	140 (68.6)	47	93		
Women	64 (31.4)	28	36		
Occupation				4.239	0.644
Agricultural workers	42 (20.6)	16	26		
Jobless	87 (42.6)	36	51		
Retiree	33 (16.2)	10	23		
Outdoor worker	27 (13.2)	10	17		
Office worker	6 (2.9)	1	5		
Self-employed person	3 (1.5)	0	3		
Student	6 (2.9)	2	4		
Surgery				15.149	**0.002**
PKP	97 (47.5)	25	72		
DALK	50 (24.5)	24	26		
ALK	53 (26.0)	22	31		
DSEK	4 (2)	4	0		
Source of grafts				0.007	0.933
Donor Corneas	179 (87.7)	66	113		
Bioengineered Corneas	25 (12.3)	9	16		
Etiology				9.401	0.310
Infectious corneal disease	89 (43.6)	32	57		
Trauma	44 (21.6)	15	29		
Keratoconus	9 (4.4)	3	6		
Corneal degeneration and congenital disease	9 (4.4)	6	3		
Immune corneal disease	12 (5.9)	4	8		
Regraft	14 (6.9)	2	12		
Endothelial dysfunction	8 (3.9)	5	3		
Corneal mass	3 (1.5)	1	2		
Unknown	16 (7.8)	7	9		
Corneal vascularization				4.755	**0.029**
Non-vascularization	160 (78.4)	65	95		
vascularization	44 (21.6)	10	34		

Non-CGR, Non-corneal graft rejection; CGR, corneal graft rejection. The meaning of the bold values indicated *p* < 0.05.

**Table 3:** Logistic regression analysis of the effect of the surgical procedures on CGR.

		Univariate analysis			Multivariate analysis	
	*p*	OR	(%95CI)	*p*	OR	(%95CI)
Age	0.869	1.002	0.982–1.022	0.935	1.001	0.978–1.024
Gender	0.100	0.667	0.408–1.074	0.199	0.714	0.427–1.194
Etiology						
Fungal	0.925	1.049	0.646–1.688	0.470	1.465	0.520–4.124
Bacterial	0.273	2.055	0.738–5.727	0.237	2.257	0.586–8.696
Amebic	0.992	0.001	0.001–0.002	0.997	0.001	0.001–0.002
Mechanical injury	0.446	0.629	0.283–1.295	0.679	0.782	0.243–2.511
Chemical injury	0.141	6.079	0.769–123.639	0.117	7.041	0.615–80.602
Thermal injury	0.390	2.018	0.469–8.674	0.244	2.692	0.510–14.227
Keratoconus	0.390	2.018	0.469–8.674	0.223	2.942	0.520–16.660
Corneal mass	1.000	0.991	0.046–10.453	0.633	1.889	0.139–25.700
Congenital	0.302	0.443	0.100–1.409	0.552	0.616	0.125–3.044
Corneal degeneration	0.986	0.001	0.001–0.002	0.994	0.001	0.001–0.002
Mooren’s ulcer	0.530	0.559	0.082–2.357	0.983	1.020	0.165–6.304
Immune	0.237	2.545	0.661–10.454	0.167	3.051	0.627–14.832
Corneal perforation	0.896	1.067	0.545–2.023	0.637	1.295	0.443–3.791
Regraft	0.419	1.616	0.695–3.672	0.588	1.376	0.434–4.364
Endothelial dysfunction	0.073	0.207	0.033–0.733	0.361	0.438	0.075–2.568
Bullous keratopathy	1.000	0.991	0.046–10.453	0.988	0.990	0.032–3.212
Corneal vascularization	0.288	1.333	0.779–2.254	0.716	1.131	0.581–2.202
Surgery						
DSEK	0.982	0.001	0.001–0.002	0.983	0.001	0.001–0.002
DALK	0.801	1.088	0.565–2.115	0.962	0.983	0.490–1.973
PKP	**0.024**	1.953	1.103–3.546	**0.046**	1.958	1.013–3.786

The meaning of the bold values indicated *p* < 0.05.

**Table 4:** Logistic regression analysis of the effect of preoperative CNV on CGR.

		Univariate analysis			Multivariate analysis	
	*p*	OR	(%95CI)	*p*	OR	(%95CI)
Age	0.092	1.038	0.995–1.085	0.077	1.040	0.996–1.086
Gender	0.380	0.629	0.200–1.669	0.265	0.548	0.190–1.579
Etiology						
Fungal	0.890	1.120	0.262–7.700			
Bacterial	0.957	0.933	0.041–10.536			
Amebic	0.999	0.001	0.001–0.002			
Mechanical injury	0.767	0.737	0.084–6.445			
Chemical injury	0.997	0.001	0.001–0.002			
Thermal injury	0.996	0.001	0.001–0.002			
Keratoconus	0.996	0.001	0.001–0.002			
Corneal mass	0.998	0.001	0.001–0.002			
Congenital	0.995	0.001	0.001–0.002			
Corneal degeneration	0.998	0.001	0.001–0.002			
Mooren’s ulcer	0.996	0.001	0.001–0.002			
Immune	0.996	0.001	0.001–0.002			
Corneal perforation	0.980	0.977	0.153–7.769			
Regraft	0.283	2.667	0.474–20.565			
Endothelial dysfunction	0.994	0.001	0.001–0.002			
Bullous keratopathy	0.998	0.001	0.001–0.002			
Corneal vascularization	**0.044**	2.607	0.986–6.562	**0.046**	2.608	1.016–6.695

The meaning of the bold values indicated *p* < 0.05.

### Analysis of the factors associated with preoperative CNV

It was found that preoperative corneal vascularization was associated with a greater risk of corneal graft rejection in our study. Corneal neovascularization was observed using a slit-lamp anterior segment imaging system (TOPCON, Japan). In the univariate analysis, we found that viral infections were more likely to result in neovascularization (*p* < 0.001, OR = 16.871, 95%CI 7.210–44.423) compared to other etiologies. The factors of fungal infections (*p* < 0.001, OR = 0.018), bacterial infections (*p* < 0.001, OR = 0.043), mechanical injury (*p* < 0.001, OR = 0.034), thermal injury (*p* = 0.045, OR = 0.183), congenital diseases (*p* < 0.001, OR = 0.020), immune corneal diseases (*p* = 0.023, OR = 0.152), corneal perforation (*p* < 0.001, OR = 0.085), regraft (*p* = 0.003, OR = 0.171), and endothelial dysfunction (*p* < 0.001, OR = 0.054) were considered to be less threatening in inducing CNV. The difference remained statistically significant in the multivariate analysis (*p* < 0.001, OR = 17.432, 95%CI 7.050–43.103), which excluded the influences of age and gender (Table 5).

**Table 5:** Logistic regression analysis of the effect of the corneal transplant etiology on preoperative CNV.

		Univariate analysis			Multivariate analysis	
	*p*	OR	(%95CI)	*p*	OR	(%95CI)
Age	0.751	1.004	0.981–1.027	0.671	1.005	0.981–1.030
Gender	0.907	1.033	0.597–1.756	0.547	0.830	0.452–1.524
Etiology						
Fungal	**<0.001**	0.018	0.005–0.054			
Bacterial	**<0.001**	0.043	0.006–0.202			
Amebic	0.994	0.001	0.001–0.002			
Mechanical injury	**<0.001**	0.034	0.008–0.117			
Chemical injury	0.941	0.913	0.098–20.089			
Thermal injury	**0.045**	0.183	0.031–0.926			
Keratoconus	0.983	0.001	0.001–0.002			
Corneal mass	0.147	0.152	0.006–1.815			
Congenital	**<0.001**	0.020	0.001–0.126			
Corneal degeneration	0.702	0.609	0.050–14.246			
Mooren’s ulcer	0.077	0.243	0.047–1.152			
Immune	**0.023**	0.152	0.026–0.727			
Corneal perforation	**<0.001**	0.085	0.026–0.242			
Regraft	**0.003**	0.171	0.050–0.534			
Endothelial dysfunction	**<0.001**	0.054	0.010–0.213			
Bullous keratopathy	0.147	0.152	0.006–1.815			
Virus	**<0.001**	16.871	7.210–44.423	**<0.001**	17.432	7.050–43.103

The meaning of the bold values indicated *p* < 0.05.

## Discussion

The retrospective analysis of the 394 patients who underwent the keratoplasty procedure in this study showed that infectious keratitis was the main etiology of corneal blindness in the southern Liaoning region. The etiology of corneal blindness varied by region and ethnicity, and in this study, fungal keratitis was the leading cause of blindness, which was more commonly caused by ocular trauma sustained during agricultural work. However, corneal contact lenses were reported to be the leading cause of infectious keratitis in developed countries (6). PKP was the preferred modality for repeat keratoplasty and corneal infections (7, 8). Barut Selver et al. (9) found that late endothelial failure (36.9%) was the most common reason for failed grafts, followed by allograft rejection (17.4%) and graft infection (14.1%), which constituted the next most frequent causes. The higher risk of graft rejection associated with PKP in our study might have been linked to the high prevalence of fungal keratitis, where the PKP procedure could completely remove the fungal infection and prevent secondary grafting infection. Moreover, DALK is associated with a relatively low rejection rate in component keratoplasty, likely due to the immunogenicity of the stroma (10). Another explanation is that the DALK procedure preserves the recipient’s corneal endothelial cell layer, which could decrease the rate of graft rejection due to endothelial dysfunction (11). The low risk of graft rejection with DALK (*p* = 0.801, OR = 1.088) was observed in our study, which might be attributed to the technical advantages of DALK. However, the specific details should be further explored in future research.

Corneal graft rejection was influenced by various factors, such as the host, donor, and surgical procedure, which could manifest as epithelial rejection lines and subepithelial infiltration, stromal infiltration and haze, endothelial Khodadoust lines, anterior chamber cells, and corneal edema (12). In our research, the patients received standardized postoperative management after the corneal transplantation, including regular postoperative follow-up, early administration of moderate-to-high concentration glucocorticoids (1% prednisolone acetate), and later administration of low-concentration glucocorticoids (0.1% fluorometholone) and immunosuppressants (1% cyclosporine A and tacrolimus). Stromal edema and haze, graft dissolution, neovascularization, recurrent infection, and epithelial defects were observed during the postoperative follow-up. CNV was a risk factor for corneal graft rejection, which is in accordance with the findings of Sibley et al. (2), who reported that the higher the number of quadrants in which neovascularization had accumulated, the greater the risk of rejection. The OR of CNV for CGR was 2.607, which is in line with the findings of a meta-analysis of 24,944 cases by Bachmann (13), who reported an OR of 1.32 for CGR due to pathological neovascularization. Therefore, these results suggest that preoperative management of vascularization could have a crucial positive effect on the prognosis of keratoplasty. Bevacizumab (14, 15) and Rapamycin (16) have been shown to effectively interfere with CNV through a subconjunctival injection before keratoplasty. In addition, corneal collagen cross-linking has been used to inhibit neovascularization, possibly by strengthening the stiffness of the corneal stroma (17, 18). Rangu et al. (19) applied mitomycin C neovascularization embolization before PKP in patients with herpes simplex keratitis. The Boston Type I artificial cornea has been used in high-risk keratoplasty, such as in cases of deep corneal stromal vascularization, to minimize the rate of graft rejection (20). However, there was no evidence to confirm that this type of graft had an effect on graft rejection (*p* = 0.933) in our research. Except for vascularization, TNF-*α* and interleukin-1, 6 (21), released by immune T cells and helper T cells, as well as the function of HIF-1α (22), were involved in the rejection. These results suggest that we should further investigate the pathological mechanisms of neovascularization and explore additional targets.

Among these cases of graft rejection, we found that viral infections were more likely to trigger neovascularization. When viral keratitis requires treatment with keratoplasty, it is likely due to the pathological cornea being in a state of fibrosis, neovascularization, and inflammation. The pathogenesis of HSK is complex and includes: (1) HSV-1 promoting the infiltration of CD4^+^ T cells, macrophages, and neutrophils, which release pro-inflammatory and pro-angiogenic factors to induce angiogenesis (23); (2) the cytopathic effects indirectly caused by HSV-1 (24); and (3) the regulation of the extracellular matrix, as well as pro-angiogenic factors other than vascular endothelial growth factor, such as interleukin-6, fibroblast growth factor-2, and matrix metalloproteinase-9 (29). Recent research has suggested that the reduction of immune cells (T cells and neutrophils) would not inhibit neovascularization (25). Therefore, the process of HSK-related neovascularization may be related to the dysfunction of corneal resident cells (corneal epithelial cells, corneal stromal cells), rather than being immune cell-mediated. Emerging histological technologies may be beneficial for studying disease mechanisms, such as single-cell RNA sequencing (26) and spatial transcriptomics (27), which could explore the molecular mechanisms of CNV and CGR in terms of cells, genes, and the spatial locations of tissues. This is likely to be a crucial breakthrough point for preoperative vascularization interventions. Certainly, interventions for immune rejection of corneal transplants should focus on the dynamics of the ocular surface microenvironment in addition to the etiology of the corneal graft. For example, tear biomarkers, such as interleukin-1β, interferon-*γ*, and interleukin-10, were thought to signal corneal graft rejection (28).

The limitations of the study primarily included an insufficient sample size. Our research team should further expand the sample size and improve the perioperative management of keratoplasty. In addition, the diagnosis of CNV and CGR was completed by slit lamp microscopy, which is highly subjective. Therefore, the depth and quadrant of CNV were not evaluated to prevent research errors. More advanced, objective analysis software could be applied to assess CNV.

In conclusion, this retrospective study analyzed the risk factors for CGR in the southern Liaoning region over the past 5 years and showed that PKP and CNV were risk factors for the rejection. Whether for patients or surgeons, this suggests that aggressive management and intervention of neovascularization preoperatively may significantly improve the survival of corneal implants. Moreover, our study found that viral infections were more likely to induce CNV, but this should be further confirmed by larger clinical studies. Meanwhile, the molecular mechanism of neovascularization caused by viral infections should be further explored through fundamental research. This became the primary focus of our research team, as it is crucial to inhibit and decrease neovascularization to increase the efficiency of corneal transplantation, reduce the prevalence of corneal blindness, and reduce the social burden of vision health in the southern Liaoning region.

## Data Availability

The original contributions presented in the study are included in the article/Supplementary material, further inquiries can be directed to the corresponding author.
