# Supplementary material for: Analysis of the relevant factors for corneal graft rejection in the southern Liaoning region from 2019 to 2023
Source: Front Med (Lausanne). 2025 Jan 8;11:1517198. doi: 10.3389/fmed.2024.1517198 (PMC11750857; doi:10.3389/fmed.2024.1517198)
Supplement: Supplementary file 1 [file Table_1.docx]

The collinearity check of multivariate regression analysis is presented as supplementary data.

| **Variable** | **GVIF** | **Df** | **Standardized VIF** |
| --- | --- | --- | --- |
| Age | 1.228279 | 1 | 1.108278 |
| Gender | 1.045620 | 1 | 1.022555 |
| Surgery Method | 2.795956 | 3 | 1.186921 |
| Disease | 4.724871 | 16 | 1.049723 |
| CNV | 1.424072 | 1 | 1.193345 |

| **Variable** | **GVIF** |
| --- | --- |
| Age | 1.016802 |
| Gender | 1.018342 |
| CNV | 1.001622 |

| **Variable** | **GVIF** |
| --- | --- |
| Age | 1.013112 |
| Gender | 1.028483 |
| Virus | 1.017158 |
